# Combination of Niclosamide and Pirfenidone Alleviates Pulmonary Fibrosis by Inhibiting Oxidative Stress and MAPK/Nf-κB and STATs Regulated Genes

**DOI:** 10.3390/ph16050697

**Published:** 2023-05-04

**Authors:** Hanaa Wanas, Hossein M. Elbadawy, Mohannad A. Almikhlafi, Amany E. Hamoud, Eid N. Ali, Amr M. Galal

**Affiliations:** 1 Department of Pharmacology and Toxicology, College of Pharmacy, Taibah University, Madinah 41477, Saudi Arabia; 2 Department of Medical Pharmacology, Faculty of Medicine, Cairo University, Cairo 11956, Egypt; 3 Department of Anatomy and Embryology, Faculty of Medicine, Cairo University, Cairo 11956, Egypt; 4 Department of Anatomy, Faculty of Medicine, Taibah University, Madinah 41477, Saudi Arabia

**Keywords:** lung fibrosis, niclosamide, pirfenidone, bleomycin

## Abstract

The pathogenesis of pulmonary fibrosis (PF) is extremely complex and involves numerous intersecting pathways. The successful management of PF may require combining multiple agents. There is a growing body of evidence that suggests the potential benefits of niclosamide (NCL), an FDA-approved anthelminthic drug, in targeting different fibrogenesis molecules. This study aimed at investigating the anti-fibrotic potential of NCL alone and in combination with pirfenidone (PRF), an approved drug for PF, in a bleomycin (BLM) induced PF experimental model. PF was induced in rats by intratracheal BLM administration. The effect of NCL and PRF individually and in combination on different histological and biochemical parameters of fibrosis was investigated. Results revealed that NCL and PRF individually and in combination alleviated the histopathological changes, extracellular matrix deposition and myofibroblastic activation induced by BLM. NCL and PRF either individually or in combination inhibited the oxidative stress and subsequent pathways. They modulated the process of fibrogenesis by inhibiting MAPK/NF-κB and downstream cytokines. They inhibited STATs and downstream survival-related genes including BCL-2, VEGF, HIF-α and IL-6. Combining both drugs showed significant improvement in the tested markers in comparison to the monotherapy. NCL, therefore, has a potential synergistic effect with PRF in reducing the severity of PF.

## 1. Introduction

Pulmonary fibrosis (PF) can be a consequence of lung injury due to irradiation, chemotherapy, exposure to environmental and occupational pollution, or due to unknown etiology as in idiopathic pulmonary fibrosis (IPF) [1]. Exposure to severe infection can also eventually lead to PF as encountered with severe SARS-CoV-2 (COVID-19) infections [2]. PF is a complex multidisciplinary process that is regulated by multiple parallel and interconnecting pathways. It represents a significant cause of morbidity and mortality worldwide [3].

Management of PF is still highly debatable, and no effective curative treatment has been established yet. The current treatment options for PF are pirfenidone (PRF) and nintedanib, the only two drugs approved by the United States Food and Drug Administration (FDA). The mechanism of PRF is poorly understood, while nintedanib is an inhibitor of tyrosine kinase. They can slow the progression of the disease, but they cannot reverse the already-developed fibrosis. Therefore, there is still an inevitable need to explore new therapeutic approaches for PF that can improve the efficacy of the treatment.

Persistent aberrant activation and senescence of alveolar epithelial cells (AECs) stimulate them to secrete different fibrinogenic cytokines that are collectively known as senescence-associated secretory phenotype (SASP) factors. These factors play an important role in the activation and enrollment of myofibroblasts from different sources including resident mesenchymal cells, circulating fibrocytes and the epithelial-mesenchymal transition (EMT) [1]. Activated myofibroblasts exhibit a stressed and senescent phenotype, including resistance to apoptosis and secretion of an excessive and abnormal extracellular matrix (ECM) that eventually leads to damage of the basement membrane, epithelial cell death and fibrotic scaring [4]. Nuclear factor-κB (NF-κB) pathway activation and activation of the Janus kinase (JAK)-STAT pathway have been found to lead to senescence and the SASP [5]. In addition, mitochondrial dysfunction may also play an important role in the pathogenesis of fibrosis. Dysfunctional mitochondria induce fibrotic remodeling and may contribute to senescence, SASP, elevated reactive oxygen species (ROS) and activation of apoptotic and inflammatory pathways [5,6].

Niclosamide (NCL), an FDA-approved oral anthelminthic drug, showed the potential of inhibiting mitochondrial oxidative phosphorylation [7]. Previous data from the literature demonstrated the effect of NCL in reducing vascular remodeling by inhibiting STAT3 and several key inflammatory cytokines. NCL showed the ability to reduce the expression of the transforming growth factor B1 (TGF-β1), hypoxia-inducible factor 1α (HIF) and vimentin, a mesenchymal marker, along with reduced EMT [8,9]. In addition, NCL decreased the growth rate and progression of endometriosis-like lesions and inhibited STAT3 and NF-κB pathways [10]. Further, this multiple pathway inhibitor is found to play an important role in alleviating TGF-β1 induced pro-fibrotic effects in some of the fibrotic diseases including renal and skin fibrosis [11,12,13]. Moreover, computational biological studies predicted NCL to show considerable anti-fibrotic effects in ameliorating PF [12].

Based on the complexity of the pathogenesis of PF, single-agent therapies are likely to only have a moderate effect. In this context, multidrug approaches that target different molecules of the fibrinogenic pathways are likely to be more effective. Here, it is hypothesized that PRF, one of the two approved drugs for PF, may exhibit synergy in inhibiting fibrogenesis when combined with other drugs that can target different fibrogenesis pathways. Finally, bleomycin (BLM) is a chemotherapeutic drug that can produce histopathological and biochemical changes in rat lungs similar to human PF [14]. BLM causes single- and double-stranded DNA breaks and produces a series of ROS [15,16,17]. This resembles pathological changes in PF and is therefore a popular model for PF.

The present study aims at investigating the anti-fibrotic potential of NCL alone and in combination with PRF in BLM induced in vivo PF experimental model. The effect of NCL and PRF individually and in combination on different histological and biochemical parameters of fibrosis was investigated. The effect of combining both drugs was compared to the effect of each individual drug to explore the supposed synergistic effect.

## 2. Results

### 2.1. Amelioration of Lung Pathohistology

**Figure 1:**
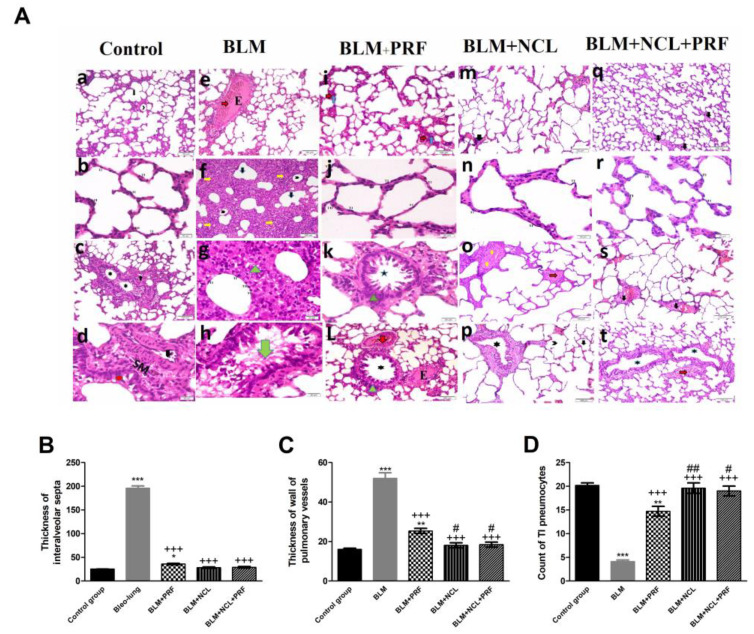
Hematoxylin and eosin staining and quantification parameters. (**A**) H&E stained sections of the lung of the control group (**a**–**d**) showing (**a**) alveoli (arrowhead) and sacs (arrow) ×100; (**b**) type1 (TI) and type2 (TII) pneumocytes ×400; (**c**) bronchiole (star) ×100; (**d**) bronchiolar lining (circle) and smooth muscles (SM) of the bronchiolar vessel (arrow) ×400; (**e**–**h**) BLM group showing (**e**) congestion (red arrow) and exudate “E” ×100; (**f**) thickened interalveolar septa (IAS) (yellow arrows), collapsed alveoli (arrowhead) and alveolar sacs (black arrow) ×100; (**g**) mononuclear cellular infiltration (triangle) ×400; (**h**) disruption of bronchiolar lining and exfoliation of cellular remnants (arrow) bronchiole ×400; (**i**–**L**) BLM + PRF group (**i**) thickened IAS septa (blue arrow) congested pulmonary vessels (red arrow) ×100; (**j**) (TI) and (TII) pneumocytes ×400; (**k**) bronchiole (star) minimal cellular infiltration (triangle) ×100; (**L**) bronchiole (star) cellular infiltration (triangle), congested (red arrow) vessel and exudate “E” ×400; (**m**–**p**) BLM + NCL group showing apparently normal alveolar sacs, alveoli and pneumocyte (**m**) thickened IAS (arrow) ×100; (**o**) congested pulmonary vessel (red arrow) and thickened vessel wall (yellow arrow) ×100; (**q**–**t**) BLM + NCL + PRF showing (**q**) mild thickening of IAS (arrow) ×100; (**s**) few thickened IAS and pulmonary vessels with thickened walls (black arrow) ×100; and (**t**) bronchioles (star) and congestion (red arrow) ×100. (**B**) shows the average thickness of the interalveolar septa. (**C**) shows the quantification of the thickness of the wall of pulmonary vessels, while (**D**) shows the count of type 1 (TI) pneumocytes. Significance vs. controls (* *p* < 0.05; ** *p* < 0.01; *** *p* < 0.001); significance vs. BLM group (+++ *p* < 0.001); significance vs. BLM + PRF (# *p* < 0.05; ## *p* < 0.01) (one-way ANOVA, followed by Tukey’s multiple range post-tests).

By histological examination of stained sections from different groups, lung sections from the BLM group showed an obviously distended pulmonary vessel, partially congested and containing exudates, in addition to marked thickening of the interalveolar septa around collapsed alveoli and alveolar sacs. Additionally, mononuclear cellular infiltration in the thickened interalveolar septa between alveoli lined by a few type1 (TI) and type2 (TII) pneumocytes was clearly noted. On the other hand, these changes were slightly ameliorated in the BLM + PRF group, where lungs showed few thickened interalveolar septa containing congested pulmonary vessels surrounded by alveoli and alveolar sacs, minimal cellular infiltration around the bronchiole as well as congestion and exudation of bronchiolar vessels. Minimal thickening of the interalveolar septa was clearly noted in the BLM + NCL and BLM + NCL + PRF groups with further improvement in the BLM + NCL + PRF group and restoration of the normal architecture in the alveoli, alveolar sac and bronchioles (Figure 1).

### 2.2. Effect on Extracellular Matrix Deposition

**Figure 2:**
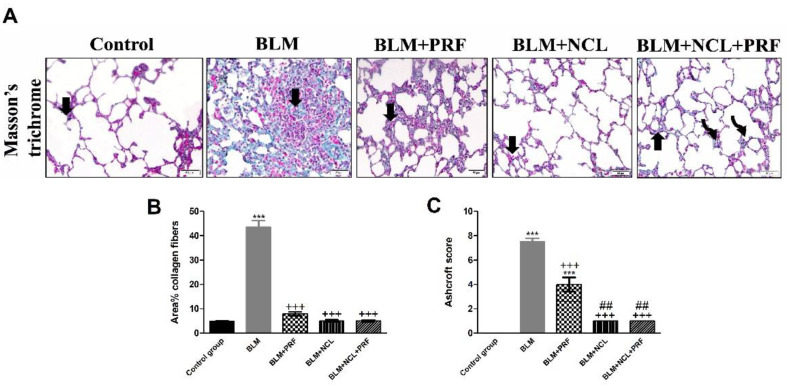
Masson’s trichrome staining and quantification of collagen fibers percentage. (**A**) Lung sections stained with Masson’s trichrome ×200 showing minimal collagen fibers (arrow) in IAS in the control group. In the BLM group, extensive collagen fibers (arrow) in IAS; in the BLM + PRF group, dense collagen fibers (arrow) in some IAS; in the BLM + NCL group, fine collagen fibers (arrow) and dense fibers in few IAS (arrow); and in the BLM + NCL + PRF group, fine collagen fibers (arrow) and dense fibers (curved arrow) in a few IAS. (**B**) Histological scoring of area % collagen fibers. (**C**) Ashcroft score. Significance vs. controls (*** *p* < 0.001); significance vs. BLM group (+++ *p* < 0.001); significance vs. BLM + PRF (## *p* < 0.01) (one-way ANOVA, followed by Tukey’s multiple range post-tests) (n = 6).

Masson’s trichrome staining was used to examine changes in the content of collagen, one of the main constituents of the extracellular matrix (ECM) [18]. In the BLM-treated group, extensive collagen fibers were found in the interalveolar septa, and dense collagen fibers were found in the BLM + PRF-treated group. This was reversed in the BLM + NCL and BLM + NCL + PRF groups. The mean percentage area of collagen fibers was significantly higher in the BLM group versus other the treated groups, while significant decreases were noted in the BLM + NCL and BLM + NCL + PRF groups, while no statistically significant differences were found between the BLM + NCL and BLM + NCL + PRF groups (Figure 2).

The Ashcroft score was evaluated to grade fibrosis in different groups. The Ashcroft score showed a significant increase after treatment with BLM. This increase was reversed in PRF and maximum reduction was seen in NCL and NCL + PRF groups (Figure 2C).

### 2.3. Immunostaining and Quantification of α-SMA and Caspase-3

**Figure 3:**
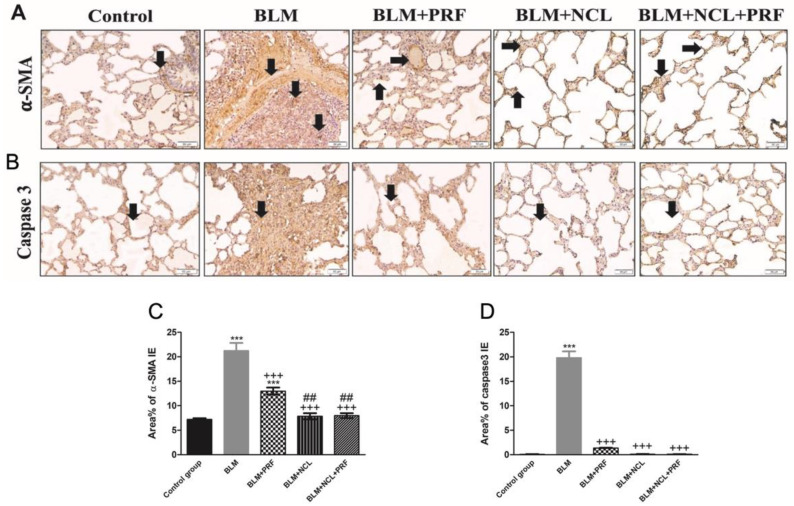
Apoptosis and myofibroblast activation in the lung section. (**A**) In α-SMA immunostaining ×200, the control rat shows +ve cells (arrows) in the smooth muscles of a bronchiole. In the BLM group, multiple +ve cells (arrow) in IAS. In the BLM + PRF group, +ve cells (arrows) in the smooth muscles of a vessel and few +ve cells in IAS. In the BLM + NCL group, few +ve cells (arrows) in IAS. The BLM + NCL + PRF group has few +ve cells (arrows) in IAS. (**B**) Caspase-3 immunostaining ×200 showing that the control rat has few +ve cells (arrows). The BLM group has multiple +ve cells (arrows) in the lining of the alveoli and IAS. The BLM + PRF group contains some +ve cells (arrows) in the lining of the alveoli and in IAS. In BML + NCL, few +ve cells (arrow) in the lining of the alveoli and few IAS. In BLM + PRF + NCL, few +ve cells (arrow) in the lining of the alveoli and few IAS. (**C**) Quantification of α-SMA. (**D**) Quantification of Caspase-3. Significance vs. controls (*** *p* < 0.001); significance vs. BLM group (+++ *p* < 0.001); significance vs. BLM + PRF (## *p* < 0.01) (one-way ANOVA, followed by Tukey’s multiple range post-tests).

Alpha smooth muscle actin (α-SMA) was used as a marker of myofibroblast activation [19]. Lung sections showed multiple positive cells in the interalveolar septa in the BLM group, with significantly fewer positive cells in the interalveolar septa in both the BLM + NCL group and the BLM + NCL + PRF groups (Figure 3A,C).

The apoptosis marker, caspase-3, was quantified in different groups. In the BLM group, multiple positive cells were found in the lining of the alveoli and in the interalveolar septa. Caspase-3 detection was significantly decreased in the BLM + PRF group. Furthermore, a marked reduction in the positivity of caspase-3 was recorded in the BML + NCL and BLM + PRF + NCL groups (Figure 3B,D).

### 2.4. Amelioration of Oxidative Stress by NCL and PRF

**Figure 4:**
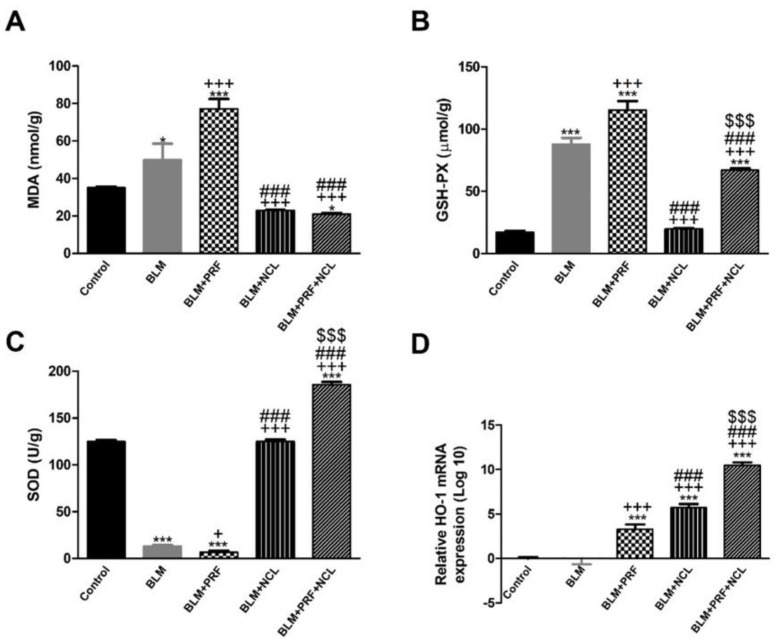
The effect of PRF and NCL on oxidative stress induced by BLM. Quantification of MDA (**A**), GSH−Px (**B**), SOD (**C**) levels and HO−1 (**D**) gene expression. Significance vs. controls (* *p* < 0.05; *** *p* < 0.001); significance vs. BLM group (+ *p* < 0.05; +++ *p* < 0.001); significance vs. BLM + PRF (### *p* < 0.001); significance vs. BLM + NCL ($$$ *p* < 0.001). (one-way ANOVA, followed by Tukey’s multiple range post-tests) (n = 6).

Intratracheal instillation of BLM resulted in a significant increase in malondialdehyde (MDA) levels in lung tissues as well as a significant decrease in glutathione peroxidase (GSH-Px) and superoxide dismutase (SOD) when compared with the control rats, whereas the administration of either NCL or NCL + PRF significantly decreased the levels of MDA and increased GSH-Px and SOD levels as compared to the BLM-only treated rats. Remarkably, the antioxidant SOD enzyme was significantly more increased in by NCL treatment than the treatment by PRF. In addition, the combination between NCL and PRF significantly increased SOD more than treatment with PRF alone. On the other hand, MDA, which is a marker of lipid peroxidation, was significantly lowered by NCL treatment and by combining NCL and PRF than the treatment with PRF alone (Figure 4A–C).

The gene expression of hemeoxygnease-1 (HO-1) was investigated. HO-1 is one of the stress response proteins that has antioxidant and anti-inflammatory properties [20]. The relative gene expression of HO-1 was notably downregulated in the BLM group in comparison to the control group. On the other hand, the expression of HO-1 was significantly upregulated in the BLM + PRF, BLM + NCL and BLM + NCL + PRF groups in comparison to the BLM group. Additionally, the up-regulation of HO-1 was significantly higher in BLM + NCL than in BLM + PRF. Finally, the up-regulation of HO-1 was significantly higher in the combination group (NCL + PRF) than in the NCL and PRF groups (Figure 4D).

### 2.5. The Effect of BLM, NCL and PRF on the Regulation of Inflammatory Mediators

**Figure 5:**
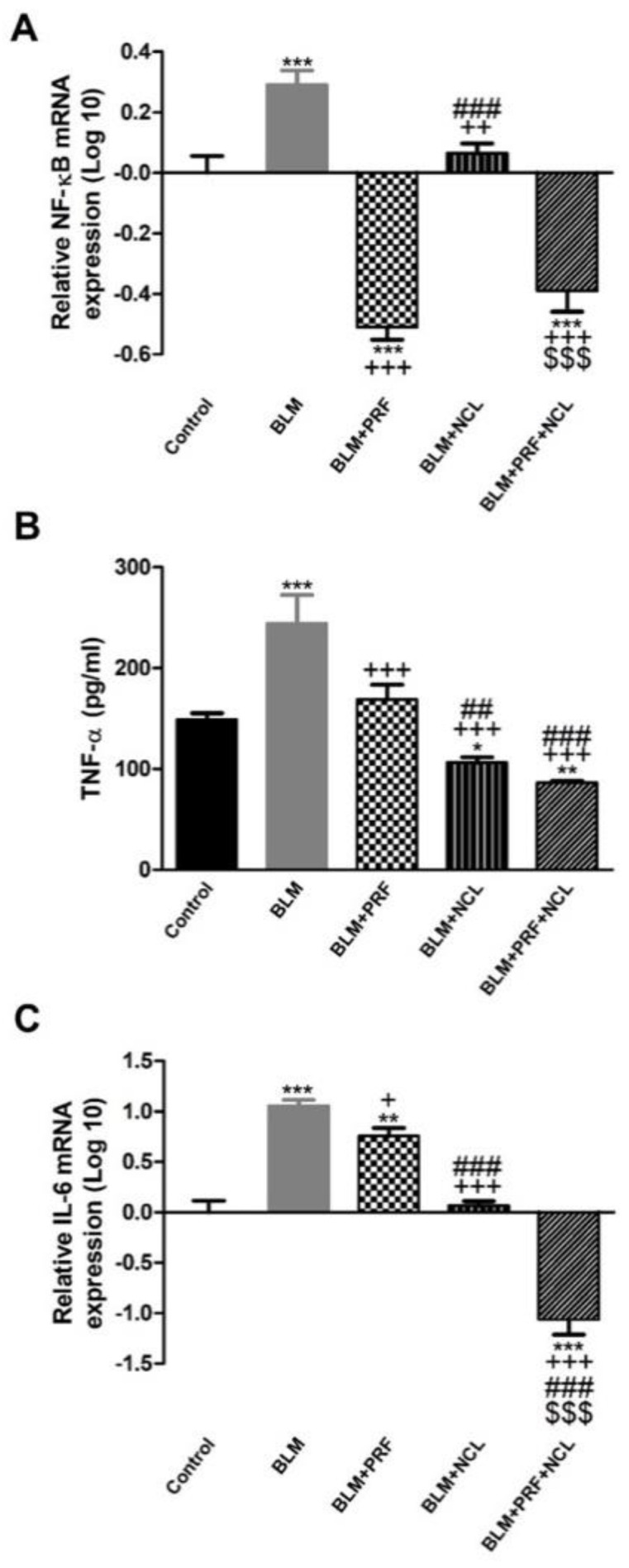
Effect of PRF and NCL on the lung inflammatory response induced by BLM. The gene expression of NF−κB (**A**), TNF−α (**B**) and IL−6 (**C**) mRNA expression in rats. Significance vs. controls (* *p* < 0.05; ** *p* < 0.01; *** *p* < 0.001); significance vs. BLM group (+ *p* < 0.05; ++ *p* < 0.01; +++ *p* < 0.001); significance vs. BLM + PRF (## *p* < 0.01; ### *p* < 0.001); significance vs. BLM + NCL ($$$ *p* < 0.001). (one-way ANOVA, followed by Tukey’s multiple range post-tests) (n = 6).

Bleomycin induced the release of inflammatory cytokines with increased mRNA levels of TNF-α in lung tissues, accompanied with surging levels of IL-6 and NF-κB gene expression. These changes were significantly reversed in the BLM + PRF, BLM + NCL and BLM + NCL + PRF groups in comparison to the BLM group. Gene expression of IL-6 was significantly lower in the BLM + NCL + PRF group than in the BLM + PRF and BLM + NCL groups. Additionally, NF-κB gene expression was significantly lower in the BLM + NCL + PRF group than in the BLM + NCL group. With regards to TNF-α, treatment with NCL and the combination between NCL and PRF significantly reduced TNF-α compared with the treatment with only PRF (Figure 5).

### 2.6. NCL and PRF Protection against Lung Fibrosis through Inhibition of STATs and MAPK Pathways

**Figure 6:**
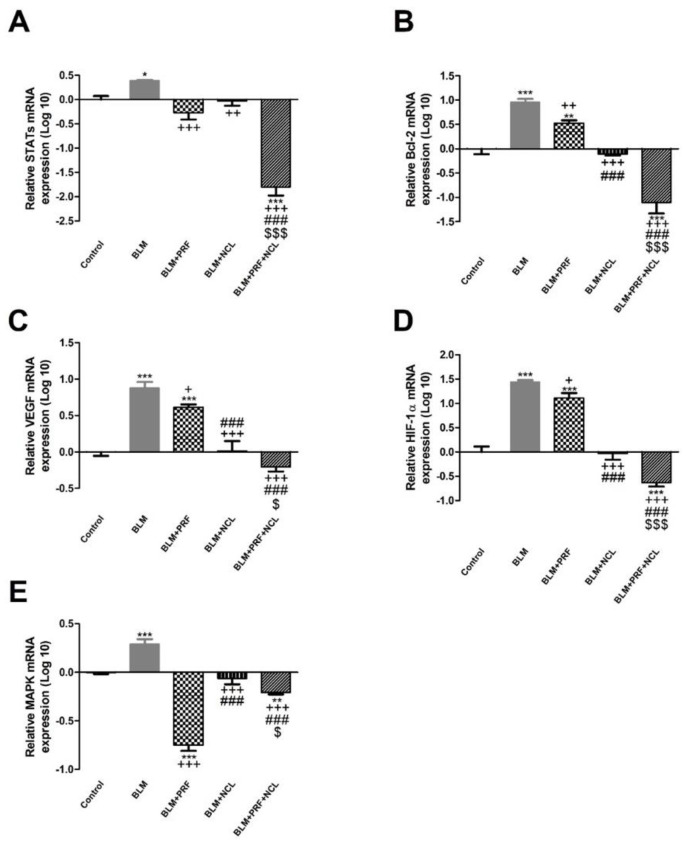
The effect of PRF and NCL on the MAPK, STATs and downstream survival-related genes. Relative gene expression changes induced by BLM in STAT (**A**), Bcl−2 (**B**), VEGF (**C**), HIF (**D**) and MAPK (**E**) mRNA expression in rat lungs are presented in bar charts on a log 10 scale. Significance vs. controls (* *p* < 0.05; ** *p* < 0.01; *** *p* < 0.001); significance vs. BLM group (+ *p* < 0.05; ++ *p* < 0.01; +++ *p* < 0.001); significance vs. BLM + PRF (### *p* < 0.001); significance vs. BLM + NCL ($ *p* < 0.05; $$$ *p* < 0.001). (one-way ANOVA, followed by Tukey’s multiple range post-tests) (n = 6).

Oxidative stress and the release of ROS in turn switch on STATs and MAPK pathways which are intersecting. STATs regulate downstream survival and proliferation genes as Bcl2, VEGF, HIF and IL-6. MAPK can upregulate the expression of NF-κB, TNF-α and IL-6 [21,22]. BLM significantly up-regulated the expression of STAT, Bcl2, VEGF, HIF and MAPK genes in comparison to the negative control group. Our results showed that the relative expression of STATs, Bcl-2, VEGF, HIF and MAPK are significantly down regulated in the PRF, NCL and NCL + NCL groups in comparison to the BLM group. Interestingly, in the combination group (NCL + PRF), all studied genes were more significantly down regulated than the NCL- and PRF-only groups (Figure 6).

## 3. Discussion

PF is a distressing lung disorder with poor prognosis and high mortality rates. Remodeling of alveolar architecture is a main feature of PF, in which normal alveolar epithelial cells are replaced by activated fibroblasts that are associated with excessive ECM production and deposition. This process eventually results in the irreversible loss of the lung’s oxygen transfer function. Oxidative stress, SASP, EMT, mitochondrial dysregulation, resistance to apoptosis with altered repair and remodeling mechanisms and exaggerated production of the extracellular matrix are considered the main features of fibrogenesis [1,2,3,5,6].

This complexity remains, posing substantial limitations to obtaining a satisfactory therapeutic outcome, especially with monotherapy treatment. Here, it was hypothesized that PRF, one of the two approved drugs for PF, may exhibit a synergistic effect in inhibiting fibrogenesis when combined with other drugs that can target different fibrogenesis pathways. NCL is an oral anthelminthic FDA-approved drug. Recently, NCL was proven to be the best candidate for effectively preventing the replication and destruction of SARS-CoV-2 after screening more than 3000 kinds of FDA-approved drugs [23]. There is a growing body of evidence on the potential benefits of NCL in targeting different molecules that participate in the fibrogenesis process [24,25,26,27,28,29,30].

Our results revealed that NCL and PRF individually and in combination attenuated the BLM-induced PF in rats. They alleviated the histological changes induced by BLM and ameliorated the ECM deposition and myofibroblastic activation induced by BLM. We demonstrated that NCL and PRF either individually or in combination inhibited the oxidative stress and subsequent pathways. NCL and PRF modulated the process of fibrogenesis by inhibiting MAPK/NF-κB and subsequent cytokines, IL-6 and TNF-α. Additionally, they inhibited STATs and subsequently regulated genes namely BCL-2, VEGF, HIF-α and IL-6.

Interestingly, the results demonstrated that combining the NCL and PRF can augment the antioxidant activity of each other. MDA, which is a marker of lipid peroxidation, was significantly lower by combining NCL with PRF than the treatment with PRF only. Additionally, the gene expression of the antioxidant marker, HO-1, was significantly more up-regulated by combining both drugs than with the individual drug treatment.

The lungs have a well-developed antioxidant defense system involving enzymes such as SOD, GSH-Px and HO-1 to maintain normal levels of ROS [31,32,33]. The imbalance between ROS production and the antioxidant defense mechanisms can not only lead to lung injury but also has a central role in the fibrogenesis process [34,35,36]. Oxidative stress promotes fibroblast migration [37], EMT senescence and the apoptosis-resistance [38]. Oxidative stress also induces angiogenesis markers such as HIF and VEGF [39] and alters the nature of the ECM [40]. Therefore, early restoration of the balance between the ROS production and the antioxidant mechanisms can help to stop the development and progression of fibrosis.

Furthermore, ROS can stimulate different signaling pathways, such as MAPK and STATs pathways [21], and activate STAT to induce the expression of inflammatory genes. STAT phosphorylation can also be regulated by MAPK and NF-κB pathways. Similarly, NF-κB and MAPK are involved in the transcription of different pro-inflammatory mediators [41,42,43]. NF-κB triggers the production of inflammatory cytokines such as TNF-α and IL-6 under the stimulation of STAT and ROS [44,45,46]. The MAPK pathway is involved in the regulation of inflammatory cytokines and chemokines and also in the activation of NF-κB [45,47,48].

We investigated the effect of NCL and PRF individually and in combination on the expression of MAPK, NF-κB and IL-6 and TNF-α levels in the lung tissues. Results demonstrated that the expression of MAPK, NF-κB and IL-6 were markedly up-regulated with BLM treatment with increased the level of TNF-α. NCL and PRF either individually or in combination significantly reversed these changes. Moreover, the combination between the two drugs exhibited synergism in inhibiting the inflammatory cytokines and the expression of MAPK.

STATs are not only involved in the inflammatory signaling. STAT family of proteins can activate the expression of many survival-related genes, including BCL2, HIF-α, VEGF and IL-6, that play an important role in interstitial lung diseases via stimulating survival, proliferation, angiogenesis and migration and resisting autophagy [22,49,50]. Insufficient autophagy in AECs lead to epithelial cell senescence, whereas in fibroblasts, lowered autophagy may induce differentiation into myofibroblasts [51].

Further, we investigated the effect of NCL and PRF individually and in combination with the expression of STATs and survival-related genes regulated by STATs. Results demonstrated that the expression of STATs, BCL2, HIF-α, VEGF and IL-6 were markedly up-regulated with BLM treatment, while their expression is markedly down-regulated with the treatment by NCL and PRF either individually or in combination. Moreover, the combination of the two drugs exhibited synergism in inhibiting STATs and other genes.

With regard to the anti-fibrotic activity of NCL, our results are in agreement with [27], where NCL reduced the serum TNF-α level in a rat model of collagen-induced arthritis. In addition, our results are in line with the results of Morin and co-workers [52], showing that NCL reduced the STAT3 pathway in a mouse model of systemic sclerosis. Moreover, our results are consistent with the observations of Braga et al. on the monocrotaline-induced pulmonary hypertension animal model [53]. They showed that NCL reduced vascular remodeling and improved right heart function via STAT3 inhibition along with the reduced expression of HIF-α, vimentin, a mesenchymal marker, along with reduced EMT. In addition, our results are consistent with the observations of [28], where NCL decreased the growth rate and progression of endometriosis-like lesions and inhibited STAT3 and NF-κB pathways in a murine endometriosis model.

Regarding the interplay between IL-6 and fibrosis in IPF patients, the results presented in the present study are in agreement with [54], where the authors showed a pro-fibrotic effect of IL-6 signaling in fibroblasts derived from patients with IPF in STAT3 dependent way. They showed that IL-6 can enhance the resistance of these cells to apoptosis by up-regulation of Bcl-2. In addition, the results of [55] showed elevated Bcl-2 in the alveolar macrophages and fibroblasts derived from mice with single intratracheal BLM installation.

Bcl-2 is a known anti-apoptotic protein. It was shown here that BLM up-regulated the expression of Bcl-2. Furthermore, we investigated the effect on caspase-3, a common marker of the intrinsic and extrinsic apoptosis pathways. BLM increased the expression of caspase-3, which is markedly decreased with the treatment by NCL and PRF either individually or in combination. These results are consistent with the results of another research group [55], which showed that BLM induces the expression of TNF and TNF receptor family genes known to induce the extrinsic apoptotic pathway, while it induces the expression of Bcl-2 that protects cells from the mitochondria-dependent intrinsic apoptosis [55].

Apoptosis of pulmonary endothelial and epithelial cells plays a pivotal role in the initiation and progression of pulmonary fibrosis [56,57,58,59]. On the other hand, decreased apoptosis of inflammatory cells and fibroblasts results in chronic inflammation, which promotes the fibrogenesis process [60]. Prolonged survival of alveolar macrophages triggers an immune response with the release of pro-fibrotic mediators [61,62]. This eventually will extend the survival time of lung fibroblasts, which is known to play a crucial role in the pathogenesis of fibrotic processes and ECM protein synthesis [63]. Consequently, the persistent existence of lung fibroblasts induces prolonged and severe interstitial lung fibrosis [64,65]. Previous studies also showed that the expression of Bcl-2 differs in different cell types, for example, lower expression of Bcl-2 was observed in alveolar epithelial cells after intratracheally instillation of BLM. While in fibroblasts and macrophages, the expression of Bcl-2 increases [54,55,66,67].

Healthy mitochondria are essential not only for their contribution to cell metabolism, but also due to their contribution in regulating energy-producing pathways. Mitochondria are strongly involved in stress responses and cell death. Additionally, mitochondria serve as the ROS’s prime source, and it was documented that their dysfunction is connected with the pathogenesis of different diseases [68]. In PF, dysfunctional mitochondria induce fibrotic remodeling and may contribute to senescence, SASP, elevated ROS and activation of apoptotic and inflammatory pathways [5,6]. Mitophagy has been implicated in myofibroblast differentiation by regulating mitochondrial ROS-mediated platelet-derived growth factor receptor (PDGFR) activation [69]. NCL induces mitochondrial uncoupling which makes the cell more susceptible to mitochondrial-dependent apoptosis [70]. By this concept, Kumar, et al. previously showed that NCL can induce apoptosis in p53-defective cancers [71]. Similarly, Qing-Shuai et al. showed that NCL inhibited cell growth and proliferation, attenuated migratory and invasive cell behaviors and promoted apoptosis [72]. Moreover, Kurita et al. showed that PRF induced autophagy/mitophagy activation and inhibited myofibroblast differentiation, reducing mitochondrial ROS [73]. The limitations of the present study are not to be addressed in measuring pulmonary function tests in different groups due to the unavailability of the required instrument. However, the histopathological changes in the different groups were sufficient.

The pathogenesis of PF is extremely complex and involves numerous parallel and intersecting pathways with interplay between a plethora of pro-inflammatory cytokines. The monotherapy strategy of PF is likely to only have a moderate effect. NCL exhibited a synergistic effect with PRF in ameliorating BLM-induced PF in rats. Combining the two drugs seems to have a high benefit in restoring the antioxidant capacity of the lung after BLM installation. In addition, the synergistic effect appeared in the ability to reduce the expression of MAPK and inflammatory cytokines. NCL can also enhance the effect of PRF in inhibiting STATs signaling and its survival-related genes. The study demonstrated the potential therapeutic benefit of combining NCL and PRF in alleviating PF and ameliorating its progression. NCL is already an approved drug that has been extensively used in clinical practice. NCL safety and tolerability have been well documented. However, the suggested benefit of adding PRF to NCL in PF management is, therefore, proposed.

## 4. Methods

### 4.1. Drugs

Bleomycin hydrochloride (bleomycin vials, 15 mg) was acquired from Nippon Kayaku Co., Ltd., Tokyo, Japan, and administered as a freshly prepared solution in normal saline for intratracheal BLM challenge experiments. Pirfenidone was obtained from Beijing Kangdini Pharmaceutical Co., Ltd., Beijing, China, and niclosamide was acquired from Sigma-Aldrich (Millipore-sigma, St. Louis, MO, USA); both were dissolved in normal saline for oral dosing.

### 4.2. Animals and Grouping

Adult male albino rats, each weighing 180–200 g, were kept under standard conditions (25 ± 2 °C room temperature; 12-h light/dark cycle with lights on at 07:30 a.m.), with free access to a standard pelleted diet and water ad libitum. All animal experiments were approved by the institutional ethics committee (No. COPTU-REC-54-20230227) and were implemented in strict accordance with approved guidelines.

Animals were randomly divided into five groups. Six rats were randomly assigned to each group: group 1 (G-I), the healthy control group was given intratracheal saline plus daily oral saline (P.O.); group 2 (G-II), the positive control disease model group (BLM) was administrated intratracheal bleomycin (BLM) then daily saline P.O; group 3 (G-III) is the pirfenidone-only group (BLM + PRF), given intratracheal BLM plus daily 50 mg/kg PRF [74] in saline P.O.; group 4 (G-IV), treated with niclosamide only (BLM + NCL), was given intratracheal BLM plus daily 100 mg/kg [75] NCL in saline P.O.; and group 5 (G-V), treated with NCL and PRF (BLM + NCL + PRF), was given intratracheal BLM plus daily 25 mg/kg PRF and 50 mg/kg NCL in saline P.O.

BLM in saline or saline alone was administered intratracheally to the rats on day 0 as described previously [14]. All rats were anesthetized with an intraperitoneal (I.P.) injection of ketamine/xylazine 100/10 mg/kg. Following anesthesia, a midline cut of the neck skin was made, and the trachea was exposed by blunt dissection. The needle of a 1 mL syringe was inserted into the trachea, and BLM dissolved in sterile saline was instilled into the rat’s lungs at a dose of 3 mg/kg [76], while rats from the control group (GpI) were instilled with an equal volume of saline. The rats were rotated immediately after injection to ensure a uniform distribution of BLM in the lungs, and then the neck skin incision was sewn. One day after the BLM challenge, oral drugs were administered intragastric (ig) once daily for 21 days. At the end of the 21 days [77], rats were euthanized, and lungs were harvested for the following experiments.

### 4.3. Histopathological Examination

Left lungs were fixed in 4% paraformaldehyde in 0.1 M PBS overnight and embedded in paraffin. Ten lung sections (5 μm each) were prepared and stained with H&E and Masson’s trichrome staining. The degree of pulmonary fibrosis was assessed in Masson’s trichrome staining based on Ashcroft’s scoring system on a numerical scale [78] in 6 fields per slide, where each field was assigned a score between 0 and 8 as follows: grade 0: normal lung, grade 1: minimal fibrous thickening of either alveolar or bronchial vessels, grade 2–3: moderate thickening of walls without distinct lung destruction, grade 4–5: increased fibrosis with definite damage to lung architecture and formation of small fibrous mass, grade 6–7: severe distortion of the structure and large fibrous areas and “honeycomb lung” grade 8: total fibrous obliteration of the field.

### 4.4. Immunohistochemical Staining

Tissue sections were de-paraffinized and rehydrated using a graded ethanol series. After antigen retrieval, eliminating endogenous peroxidase and pre-incubating in 5% BSA to block background staining, sections were incubated with rabbit polyclonal anti-α-SMA (1:100) (ab5694). Antibodies were purchased from Abcam^®^ (Cambridge, MA, USA). The color reaction was then made with an HRP-linked polymer detection system and counterstained with hematoxylin as previously described [14]. Paraffin-embedded mouse spleen tissue was used as a positive control. Negative controls were processed via omission of the primary antibodies in the automated staining protocol. Caspase-3 antibody (RB-1197-R7, Lab Vision Corporation, Fremont, CA, USA) was used for immunostaining as a marker for apoptosis. Prediluted 1ry rabbit polyclonal caspase-3 antibody (1:100) was added (0.1 mL) for 60 min. The reaction was cytoplasmic. Human tonsils served as a positive control.

### 4.5. Caspase-3 and α-SMA Immunostaining

Leica Qwin 500 LTD (Cambridge, UK) image analyzer was used to measure the following parameters: The thickness of the interalveolar septa and that of the pulmonary vessels wall. The area percentage of collagen fibers, caspase-3 and α-SMA immunoexpression (IE) were also performed. All measurements were done using 6 non-overlapping fields taken from 6 slides of different groups.

### 4.6. MDA, SOD, GSH-Px and TNF-α Measurements

The residual blood was removed from the sample by washing tissue with pre-cooled PBS buffer (0.01M, pH = 7.4). Then, the tissue was minced after weighing (0.1 g) and homogenized in 1 mL PBS. A protease inhibitor was added into the PBS with a glass homogenizer on ice. To further break the cells, the suspension underwent sonication using ultrasonic cell disrupter. The homogenates were then centrifuged for 5 min at 5000× *g* to collect the supernatant. The total protein concentration was determined using BCA total protein kit by following the manufacturer protocol (Millipore-sigma, St. Louis, MO, USA).

Malonaldehyde [79] colorimetric assay kit, catalog No: E-BC-K025-S, (Elabscience Biotechnology, Houston, TX, USA), Superoxide Dismutase [80] Assay Kit, cat no:E-BC-K020, (Elabscience Biotechnology, Houston, TX, USA). Glutathione Peroxidase (GSH-Px) Activity Assay Kit, (Elabscience Biotechnology, Houston, TX, USA), cat no: E-BC-K096-S and Rat TNF-α (Tumor Necrosis Factor Alpha) ELISA Kit, cat no: ELK1396, purchased from ELK Biotechnology, Wuhan, Hubei, China, were used according to the manufacturer’s instructions.

### 4.7. RNA Isolation and Quantitative Real-Time PCR (qRT-PCR)

Disruption and homogenization for lung tissue were performed using the Tissue Ruptor II (Qiagen, Hilden, Germany), a rotor-stator homogenizer that thoroughly disrupts and simultaneously homogenizes single tissue samples in the presence of guanidine-thiocyanate-containing lysis buffer with RNAse inhibitor. Then the mixture was collected for RNA extraction.

Following disruption and homogenization of lung tissue, samples were loaded onto RNeasy Mini spin column. Total RNA was eluted in RNase-free water. The RNA extraction and purification was performed using RNeasy blood/ tissue Mini kit (Qiagen, Hilden, Germany). The process was conducted according to the manufacturer’s protocol.

The reverse transcription step was performed by the QuantiTect Reverse Transcription Kit, cat. No: 205310, (Qiagen, Hilden, Germany). The reverse transcription master mix was prepared on ice in a total volume of 20 µL, which was composed of 1 µL of Quantitect Reverse transcriptase enzyme, 4 µL of RT buffer, 1 µL of RT primers mix and 14 µL of isolated RNA. The reaction mix was incubated for 2 min at 42 °C to remove genomic DNA, followed by 15 min incubation at 42 °C to allow for cDNA synthesis, then ending the reaction by incubation for 3 min at 95 °C to inactivate Quantiscript Reverse Transcriptase.

The gene expression level was amplified from mRNA using specific primer sequences “QuantiTect Primer Assay” for each gene, cat no: 249900 (STATs: Rn_LOC311021_1_SG, assay ID: QT00396235, MAPK: Rn_Lamtor1_1_SG, assay ID: QT00434112, and NF-KB: Rn_RGD:727889_1_SG, assay ID: QT00381227, BCL2: Rn_Bad2_1_SG, Assay ID: QT00190407, HIF-α: Rn_Hifa_1_SG, assay ID: QT00182532, IL-6: Rn_IL6_SG, assay ID: QT00176834, VEGF: Rn_VEGFβ_SG, assay ID: QT01290163 and Hem oxygenase 1: Rn_Fcho1 _SG, assay ID: QT01291129). All the primer assays were purchased from Qiagen, Hilden, Germany. The QuantiTect Primer Assay ACTβ_1_SG QuantiTect Primer Assay (β-actin) cat no: 249900, assay ID: QT00095431, was used as a housekeeper gene. All samples were analyzed using the 5 plex Rotor Gene PCR Analyzer (Qiagen, Germany). The PCR reaction mix was prepared by adding 2× QuantiTect SYBR Green PCR Master Mix, 10× QuantiTect Primer Assay, template cDNA and RNase-free water, which were thawed at room temperature (15–25 °C). Then, the reaction mix was prepared for a final volume of 18 µL per well reaction volume as follows: 10 µL of 2× QuantiTect SYBR Green PCR Master Mix, 2 µL 10× t Universal Primer, 2 µL 10× Quantitect Primer Assay and 4 µL RNase-free water. The reaction mix was mixed thoroughly but gently and dispensed appropriate volumes into the 96-well plate; then, 2 µL template cDNA was added to reach 20 µL as the final volume. Carefully, the disc was tightly sealed with a heat-sealing film.

Consequently, the real-time cycler was programmed as follows: activation step for 15 min at 95 °C for Taq DNA Polymerase activation. Three-step cycling was as follows: denaturation for 15 s at 94 °C, annealing for 30 s at 55 °C and extension for 30 s at 70 °C for 40 cycles. The expression levels were normalized to β-actin levels as a reference gene. The relative gene expression level (fold change) for APP was normalized to an internal control (β-actin) and calculated relative to the calibrator (negative control sample) using the equation 2^−ΔΔCt^ test/control. Results were finally presented in log 10 scale.

### 4.8. Data Analysis

The measurements and counts were tabulated, graphically illustrated and subjected for statistical analysis. The obtained data were analyzed using SPSS (statistical package for social sciences) version 15. The numerical data were presented as mean ± standard deviation. The statistical significance of the differences between mean values were assessed using ANOVA with multiple comparisons and Bonferroni post hoc testing. Data from MDA, GSH-Px and SOD quantification and comparisons of real time PCR results were analyzed by one-way ANOVA, followed by Tukey’s multiple range post-tests. The probability level was at 95% confidence level. A value of *p* ≤ 0.01 was considered significant.

## Data Availability

No new data were created or analyzed in this study. Data sharing is not applicable to this article.
